# Emerging roles of extracellular vesicles in oral and maxillofacial areas

**DOI:** 10.1038/s41368-024-00341-9

**Published:** 2025-02-04

**Authors:** Qianting Wang, Jiayu Sun, Haci Jiang, Mengfei Yu

**Affiliations:** Stomatology Hospital, School of Stomatology, Zhejiang University School of Medicine, Clinical Research Center for Oral Diseases of the Zhejiang Province, Key Laboratory of Oral Biomedical Research of Zhejiang Province, Cancer Center of Zhejiang University, Hangzhou, China

**Keywords:** Nanoparticles, Mechanisms of disease

## Abstract

The oral and maxillofacial region is a highly complex area composed of multiple tissue types and bears various critical functions of the human body. Diseases in this region pose significant diagnostic and management challenges; therefore, exploring new strategies for early diagnosis, targeted treatment, and tissue reconstruction is key to improving patient prognosis and quality of life. Extracellular vesicles are a group of heterogeneous lipid-bilayer membrane structures secreted by most cell types, including exosomes, microvesicles, and apoptotic bodies. Present in various body fluids and tissues, they act as messengers via the transfer of nucleic acids, proteins, and metabolites to recipient cells. To date, studies have revealed the different roles of extracellular vesicles in physiological or pathological processes, as well as applications in disease diagnosis, prognosis, and treatment. The importance and tissue specificity of the dental and maxillofacial tissues indicate that extracellular vesicles derived from this region are promising for further research. This paper reviews the published data on extracellular vesicles derived from cells, body fluids, and tissues in oral and maxillofacial regions, summarizes the latest advances in extracellular vesicles from extensive sources, and concludes with a focus on the current research progress and application prospects of engineered exosomes in oral science.

## Introduction

The oral and maxillofacial region is a complex system with several tissue types, including teeth, periodontal tissues, sense organs, skin, and mucosa. As a result, it carries various physiological functions such as pronunciation, mastication, and facial esthetics. Conditions such as infection, trauma, developmental deformities, and tumors that target this region may cause structural defects, dysfunctions, and/or adverse effects on facial appearance, as well as poor quality of life for patients. Considering the importance and complexity of the oral and maxillofacial region, there is a need for comprehensive research on physiology, development, and pathology in this region as well as innovative strategies for early diagnosis and effective treatment for the above-mentioned conditions.

Extracellular vesicles (EVs) are nanoscale membrane vesicles arising from almost all types of cells including bacteria, mammals and plants, and are abundant in numerous biological fluids, including blood, saliva, urine, and breast milk.^1^ Based on their biogenesis, EVs can be divided into exosomes, microvesicles, and apoptotic bodies (ABs).^2^ The most widely studied EVs are exosomes with a diameter of 30–150 nm, formed by the inward budding of the endosomal membrane after the fusion of multivesicular bodies with the plasma membrane.^3^ Microvesicles, measuring 100 nm to 1 μm, are a type of EV released during normal cellular activities, which originate from the plasma membrane. ABs, on the other hand, are larger (1–5 μm) and are produced during cell death, which are rarely used in research due to their large and heterogeneous particle size.^1^ The classification criteria for the subtypes of EVs have not yet been unified. According to the updated guidelines of the International Society for Extracellular Vesicles of 2018 (MISEV2018), EVs can also be classified into medium/large EVs (>200 nm) and small EVs (sEVs) (<200 nm) based on their sizes.^4^ EVs transport a range of biomolecules, including proteins, lipids, nucleic acids, and other biologically active substances, which vary according to the parent cell type and differentiation status, as well as specific stimuli within the microenvironment. Once internalized by recipient cells, EVs modulate various physiological or pathological processes, including metabolism, tissue regeneration, immune modulation, cell proliferation and differentiation, and tumor growth.^5^ Accumulating studies suggest that as “molecule carriers”, EVs can act as innovative instruments for different diagnostic and therapeutic purposes including anti-tumor therapy and drug delivery.

EVs play a key role in the development of oral and maxillofacial region. Jiang et al.^6^ have reported that exosomes might mediate epithelium-mesenchyme cross-talk through reciprocally evoking cell differentiation and matrix synthesis, thereby regulating tooth germ development. Elsewhere, Hayashi et al.^7^ observe that miRNA-rich exosomes isolated from the mesenchyme of fetal mouse submandibular glands were capable of moving to the epithelium during the formation of these glands.

The roles of EVs in oral and maxillofacial diseases have also been widely reported. In oral squamous cell carcinoma (OSCC), EVs not only regulate the tumor microenvironment (TME), facilitate tumor growth and metastasis, but also involve in tumor immune escape by modulating the immune response.^8–10^ Cancer-associated fibroblasts (CAFs) can transport highly expressed miRNAs to OSCC cells through exosomes in order to promote OSCC progression.^11^ Additionally, EVs are associated with autoimmune disorders, such as rheumatoid arthritis, Sjögren’s syndrome (SS), and systemic lupus erythematosus.^12^

Many scholars focus on the applications of EVs in oral and maxillofacial areas. The detection of EVs in biological fluids potentially offers a multicomponent diagnostic readout. Significant differences in the number, size, protein, and miRNA levels of salivary EVs between patients and healthy individuals imply that salivary EVs can act as biomarkers for diagnosing periodontitis, OSCC, SS, oral lichen planus (OLP), and other oral diseases, simultaneously indicating their progression.^13–16^ The effective exchange of cellular components via EVs provides a reference for developing EV-based therapeutics. For instance, Huang et al.^17^ discovered that human dental pulp stem cells (DPSCs)-derived EVs could play a role in dental pulp tissue regeneration.

**Fig. 1:**
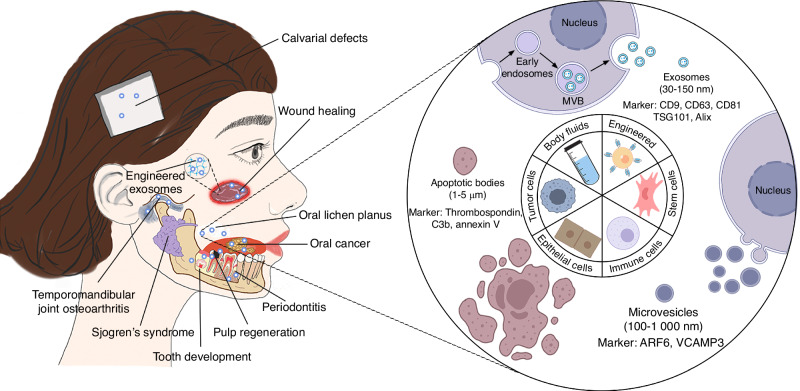
Schematic illustration of the biogenesis, sources, and roles of extracellular vesicles in oral and maxillofacial areas. Extracellular vesicles have been detected in almost all cell types and body fluids, and divided into exosomes, microvesicles, and apoptotic bodies according to their size distribution and biogenesis

**Fig. 2:**
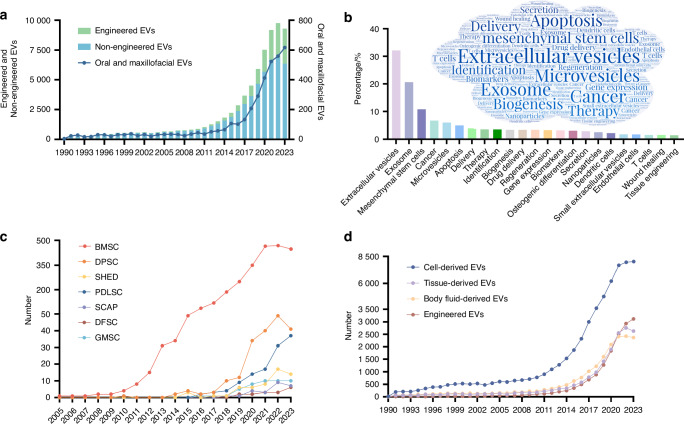
Number and keyword analysis of publications. **a** Timeline (1990–2023) of the publications referring to oral and maxillofacial EVs, engineered EVs, and non-engineered EVs according to our search strategy. **b** The frequency distribution and cloud maps of keywords in the publications referring to oral and maxillofacial EVs. **c** Timeline (2005–2023) of the publications referring to EVs derived from different oral tissue stem cells. **d** Timeline (1990–2023) of the publications referring to four different sources of EVs

Considering the rich tissue composition, the oral and maxillofacial region contains various EVs from diverse sources (Fig. 1). By transporting distinct cargos, these EVs exert diverse effects on different recipient cells (Supplementary Table 1). Therefore, understanding the characteristics and functions of EVs from different sources is essential to exploring their effects on physiological and pathological processes, as well as their application potential. This review searched the Web of Science and PubMed databases, and found a substantial rise in the number of publications referring to oral and maxillofacial EVs from 1990 to 2023 (Fig. 2). We summarize important research progress and challenges of EVs in the oral and maxillofacial region, as well as their roles in diagnosis, prognosis, and treatment. Due to the length of this article, we are unable to discuss microbial EVs, which are a significant group of EVs found in the oral and maxillofacial areas. Although the International Society for EVs has proposed EVs as a preferential term to describe all the previously mentioned types, this review uses the term “exosomes, sEVs, microvesicles, ABs, and EVs” in accordance with the original article.

## Epithelial cell-derived EVs

**Fig. 3:**
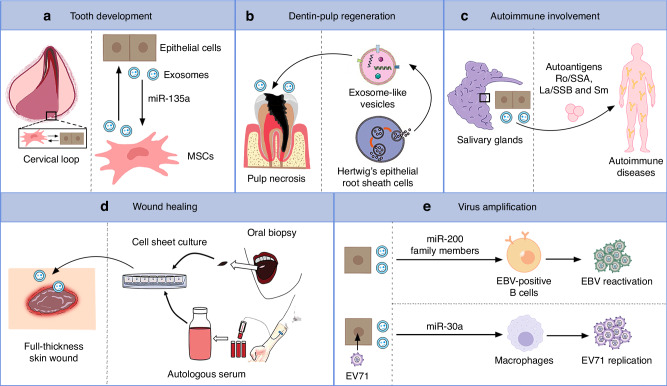
The different functions of epithelial cell-derived EVs under different conditions. **a** EVs participate in tooth development by mediating epithelial-mesenchymal cross-talk. **b** Exosome-like vesicles derived from Hertwig’s epithelial root sheath cells promote pulp-dentin regeneration.^19^ **c** Exosomes from salivary gland epithelial cells contain vital autoantigenic targets, Ro/SSA, La/SSB, and Sm, in patients with systemic rheumatic disorders such as SS and systemic lupus erythematosus. **d** Exosomes derived from clinical-grade oral mucosal epithelial cell sheets show pro-regenerative effects on skin wound healing.^20^ **e** EVs promote EBV reactivation in B cells and Enterovirus 71 replication via the transfer of different miRNAs. MSCs mesenchymal stem cells, SS Sjögren’s syndrome, EBV Epstein-Barr virus

Epithelial cell-derived EVs from different locations have diverse functional properties. Epithelial cell-derived EVs may participate as novel mediators in tooth development, which involves complex epithelial-mesenchymal interactions.^18^ As mentioned above, exosomes might mediate epithelium-mesenchyme cross-talk during tooth development^6^ (Fig. 3a). Through in vitro and in vivo experiments, Zhang et al.^19^ found that exosome-like vesicles derived from Hertwig’s epithelial root sheath cells promoted the migration, proliferation, and odontoblast differentiation of dental papilla cells, and triggered regeneration of dental pulp-dentin like tissues in a tooth root slice model (Fig. 3b). Sjöqvist et al.^20^ first explored the healing capacity of oral mucosa from an exosome perspective; they found that exosomes derived from oral mucosal epithelial cell sheets positively influenced skin wound healing due to their pro-regenerative properties (Fig. 3d).

Epithelial cell EVs are involved in diseases. Exosomes from salivary gland epithelial cells (SGECs) contain vital autoantigenic targets, Ro/SSA, La/SSB, and Sm, in patients with autoimmune diseases including SS and systemic lupus erythematosus^21^ (Fig. 3c). Epithelial cell EVs may also play a role in viral infection (Fig. 3e). For instance, oral epithelial cell-membrane vesicles contain miR-200 family members, which initiate reactivation in Epstein-Barr virus (EBV)-positive B cells, thereby promoting viral amplification and dissemination to other hosts.^22,23^ Exosomes produced by oral epithelial cells infected with Enterovirus 71 specifically pack high levels of miR-30a to the recipient macrophages and subsequently facilitate virus replication.^24^

## Immune cell-derived EVs

Immune cell-derived EVs can influence the immune response and, in turn, affect the development of multiple oral diseases. They also have a dual effect on tumor progression. Furthermore, certain EVs, especially those derived from macrophages, are implicated in the regulation of bone metabolism.

### Immunoregulation

**Fig. 4:**
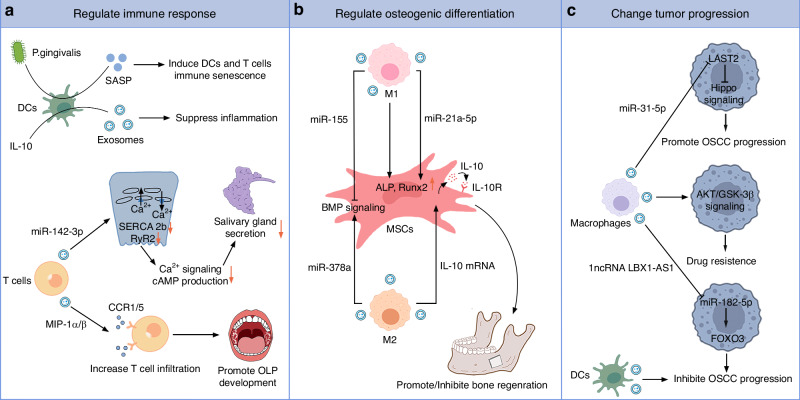
The bioactive effects of immune cell-derived EVs on osteogenesis, immune responses, and tumor progression. **a** *P. gingivalis* induced DC exosomes promote immune senescence, while IL-10-treated DC exosomes suppress inflammation. T-cell exosomes impair glandular cell function in SS, and drive the trafficking of CD8+ T cells in OLP by carrying different messages. **b** Different polarized macrophage-derived EVs carrying different cargos have a dual role in osteogenic differentiation. **c** Macrophage-derived exosomes have a two-sided effect on tumor cells while DC-derived exosomes inhibit tumor progression. MSCs mesenchymal stem cells, DCs dendritic cells, SS Sjögren’s syndrome, OLP oral lichen planus, OSCC oral squamous cell carcinoma

Extensive research has established that immunopathogenesis regulates the development and progression of oral diseases such as periodontitis, OLP, and SS (Fig. 4a). Interestingly, accumulating evidence underscores the roles of EVs from immune cells in promoting communication among different immune cell types, thereby dynamically regulating host immune response. Cutler CW and his colleagues^25–27^ revealed that *P. gingivalis* induced inflammatory exosomes from infected dendritic cells (DCs), further promoting paracrine senescence of bystander DCs and T cells, and inducing alveolar bone loss in vivo. Evidence suggests that viral infections contribute to the pathogenesis of SS. EBV-miR-BART13-3p, a specific miRNA from EBV, can be transmitted from EBV-infected B cells to SGECs through exosomes, resulting in salivary gland dysfunction.^28^ In addition, exosomes containing miR-142-3p from activated T cells disrupted mechanisms associated with a secretory function of SGECs, which are implicated as pathogenic drivers of SS.^29^ Yang et al.^30,31^ revealed that T cell-derived exosomes increased macrophage inflammatory protein synthesis, drove the trafficking of CD8+ T cells, and triggered keratinocytes apoptosis, thus participating in the pathogenesis of OLP. Immune cell-derived EVs may also represent an innovative, cell-free therapy for oral diseases, as exosomes derived from IL-10-treated DCs could inhibit collagen-induced arthritis.^32^

### Regulate osteogenic differentiation

Investigations reveal that macrophages regulate the immune response of mesenchymal stem cells (MSCs) and osteoblasts in bone regeneration, and their involvement in chronic inflammation-induced alveolar bone loss is well-documented.^33,34^ However, the functionality of macrophage-derived EVs in bone metabolism has not been thoroughly studied. Kang et al.^35^ confirmed that macrophage polarization caused different miRNA cargo in EVs, resulting in M0 and M2 EVs promoting bone regeneration while M1 EVs inhibited bone repair. In addition, a recent study revealed that M2 exosomes prevented pathological alveolar bone resorption in mice with periodontitis.^36^ However, Xia et al.^37^ reported conflicting findings, suggesting that only the medium containing M1 exosomes, rather than M0 exosomes or M2 exosomes, supported osteogenic and adipogenic differentiation. Interestingly, Liu et al.^38^ indicated that both M1 and M2 exosomes could improve the osteogenesis of bone marrow-derived mesenchymal stem cells (BMSCs). M1 exosomes were particularly effective in promoting bone formation during the initial inflammatory phase. The low oxygen levels in the gums caused by periodontitis could induce macrophage apoptosis.^39^ Researchers studied ABs released following macrophage apoptosis and found they could inhibit osteoblast differentiation and promote alveolar bone resorption in periodontitis.^40^ These studies provide a novel perspective on the complex interactions between BMSCs and macrophages (Fig. 4b).

Macrophage-derived EVs could potentially serve as a functional tool in biomaterial-based bone regeneration. Liu et al.^41^ found that EVs extracted from macrophages cultured on various mineralized collagen contributed to MSCs osteogenesis, whereas blocking their secretion significantly impaired MSCs osteogenic potential. Zhang et al.^42^ noted that exosomes derived from RAW264.7 cells could improve the osteogenic differentiation and mineralization of MC3T3-E1 cells in osseointegration around titanium implants with small-scale topography.

### Change tumor progression

Immunotherapy represents a groundbreaking approach to cancer treatment. DC vaccination has been shown to effectively stimulate the immune system against tumors in both preclinical and clinical studies. However, the number of patients who have benefited from clinical trials conducted over the past two decades is relatively limited.^43^ Numerous technical limitations associated with DC-based immunotherapy have been resolved by DC-derived exosomes. Accordingly, Phase I and II clinical trials adopting DC-derived exosomes have been conducted in advanced malignancies, demonstrating the propensity of exosomes to mediate immune responses based on T and natural killer (NK) cells in patients.^44^ However, macrophage-derived EVs may have two-sided effects on tumor cells (Fig. 4c). Ai et al.^45^ discovered that exosomal LncRNA derived from RBPJ overexpressed macrophages inhibited OSCC progression. In contrast, Yuan et al.^46^ revealed that M2 macrophage-derived exosomal miRNA could facilitate OSCC progression. Tomita et al.^47^ confirmed that macrophage-derived EVs could decrease the sensitivity of OSCC cells to chemotherapeutic drugs.

## Tumor-derived EVs

Cancer development is a dynamic process involving multiple components, including tumor cells, stromal cells, and immune cells. Tumor-derived EVs, which transmit information from tumor cells to other normal or abnormal cells, have been linked to several hallmark features of cancer. While EVs may not be the primary driver of tumors, they are important mediators of disease progression, influencing tumor growth, metastasis, the TME, and resistance to treatment.

### EVs facilitate tumor growth and metastasis

OSCC cell-derived exosomes could be absorbed by OSCC cells themselves, leading to a significant promotion of cell proliferation, migration, and invasion.^48^ Dickman et al.^8^ found that OSCC cells selectively packaged miR-142-3p into sEVs, thereby mitigating its tumor suppressive growth inhibitory effect in donor cells while improving the proangiogenic activity of recipient cells. Exosomes from highly metastatic OSCC cell lines could induce an aggressive phenotype in non-invasive counterparts by delivering miR-1246^49^ and miR-200c-3p.^50^ Kozaki KI and his colleagues examined the proteomic profile of EVs from OSCC cell line HSC-3 and found multiple overexpressed oncogenic proteins including EGFR^51^ and HSP90.^52^ In a hypoxic microenvironment, OSCC cells produced exosomes rich in miR-21, which were absorbed by normoxic cells, resulting in a prometastatic phenotype.^53^ Besides, hypoxic head and neck squamous cell carcinoma (HNSCC) cell-derived sEVs induced recipient non-hypoxic HNSCC cells invasion and stimulated premetastatic niche formation by delivering LOXL2.^54^

**Fig. 5:**
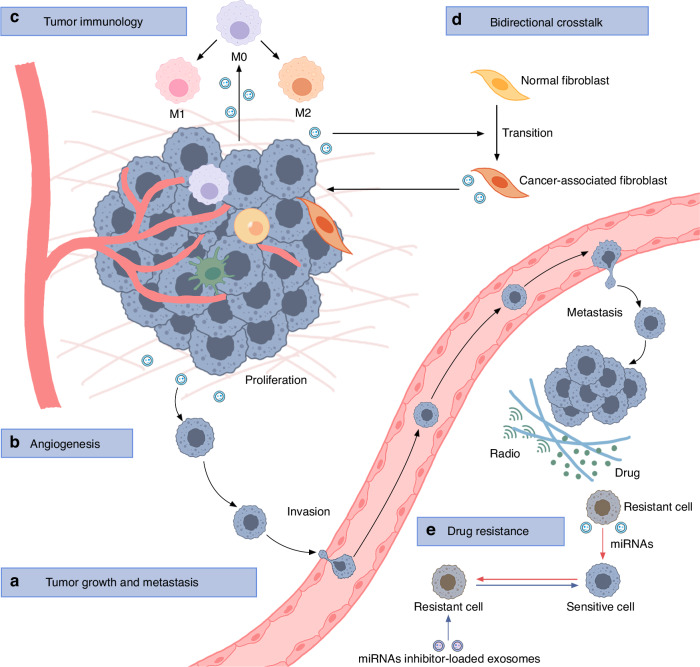
Roles and regulatory mechanism of EVs in tumor. **a** EVs improve tumor cell proliferation, migration, and invasion. **b** EVs promote angiogenesis. **c** EVs regulate tumor immunology by reprogramming macrophages, DCs, and T cells. **d** Tumor-derived EVs act as messengers in the TME by activating CAFs whereas CAF-derived EVs alter the gene expression of tumor cells. **e** EVs confer cisplatin resistance and radioresistance and can serve as targets for tumor therapy. CAFs cancer-associated-fibroblasts, TME tumor microenvironment

Apart from OSCC and HNSCC, exosomes from salivary adenoid cystic carcinoma (SACC) cell lines improve tumor cell migration and invasion by downregulating cell junction-related protein expression.^55^ EBV-positive nasopharyngeal cancer (NPC) cell-derived exosomes damaged the endothelial cell tight junction proteins and promoted endothelial-to-mesenchymal transition, thereby facilitating tumor metastasis^56^ (Fig. 5a). EVs rich in EGFR from highly metastatic NPC cells enhanced the metastatic potential of poorly metastatic NPC cells.^57^ Besides EGFR, You et al.^58^ revealed that MMP13-contained exosomes derived from NPC could promote metastasis of NPC cells. They also found a higher level of MMP13 in exosomes from hypoxic NPC cells, which further improved tumor migration and invasiveness.^59^

### EVs promote angiogenesis

Tumor-derived EVs can stimulate angiogenesis to promote tumor progression by selectively packaging miRNAs and proteins (Fig. 5b). OSCC-derived EVs delivered miRNAs to human umbilical vein endothelial cells (HUVECs) to improve cell migration, proliferation, and angiogenesis.^60,61^ OSCC-derived exosomes carrying miR-130b-3p could promote angiogenesis by downregulating PTEN expression, an anti-tumor factor.^62^ Similarly, SACC-derived exosomal miR-23b-3p also targeted PTEN to promote tumor angiogenesis and metastasis.^63^ Several other NPC-derived exosomal miRNAs are also involved in mediating angiogenesis.^64–68^ According to proteomic analysis of HNSCC sEVs, Ephrin type B receptor 2 is a candidate angiogenic protein cargo.^69^ Elsewhere, another study provided evidence for HNSCC-derived exosomal adenosine to promote angiogenesis via A2BR.^70^ Chan et al.^71^ identified differentially expressed proteins in NPC-derived exosomes using iTRAQ-based quantitative proteomics; they found that angiogenic proteins including intercellular adhesion molecule-1 (ICAM-1) and thrombospondin-1 acted as important mediators in exosome-induced angiogenesis. You Y and his colleagues^72,73^ indicated that EVs secreted by NPC cells had high levels of HAX1, which activated the FAK pathway in endothelial cells, thereby promoting angiogenesis. Unlike healthy cells, tumor cells secrete numerous nuclear exosomes.^74^ HMGB3-containing nuclear exosomes secreted by NPC cells were absorbed by HUVECs and induced angiogenesis.^75^

### EVs regulate tumor immunology

Tumor-derived exosomes may potentially alter the fate of macrophage phenotypes^76^ (Fig. 5c). Different contents, including miRNAs and proteins, have been identified in OSCC-derived exosomes to improve cell proliferation, migration, and invasion by differentially activating macrophages, either in M1 or M2 states.^9,77,78^ Among them, HPV + HNSCC exosomes are enriched in miR-9, which induces M1 polarization and increases the radiosensitivity of HNSCC.^79^ Upon activation of endoplasmic reticulum stress, OSCC cells secreted more PD-L1-enriched exosomes, which upregulated PD-L1 expression in macrophages and promoted M2 macrophage polarization.^80^ For the first time, Wang et al.^81^ demonstrated that NPC-derived exosomes, but not exosomes from normal nasopharyngeal epithelial cells, significantly induced IL-6 production from macrophages, in turn increasing the malignant behavior of NPC cells. Besides macrophages, tumor-derived EVs could also exert immunoinhibitory effects on human T cells,^82^ enhance the cytotoxicity of NK cells,^83^ and differentially reprogram human DCs.^84^ Mrizak et al.^85^ revealed for the first time that NPC-derived exosomes carry the CCL20 chemokine, which can recruit Tregs to the tumor. These exosomes induced Treg expansion, improved their suppressive functions, and encouraged the conversion of conventional CD4 + T cells into Tregs, thereby helping NPC cells to avoid detection by the immune system.

### EVs evolving in bidirectional cross-talk

Bidirectional communication between cells and their microenvironment is essential for maintaining normal tissue homeostasis and promoting tumor growth. Besides facilitating cross-talk amongst tumor cells, EVs can also influence the TME (Fig. 5d). CAFs play a key role in the metabolic and immune reprogramming of the TME.^86,87^ Tumor-derived EVs can act as messengers in the TME by activating CAFs.^88–90^ For instance, NPC-derived EVs rich in LMP1 could activate normal fibroblasts into CAFs, further promoting the proliferation, migration, and radioresistance of NPC cells via autophagy and coupling of stroma-tumor metabolism.^91^ Jiang et al.^10^ revealed that normal human gingival fibroblasts transformed their phenotype to CAFs when co-cultured with OSCC-derived microvesicles, in turn facilitating OSCC cell migration and invasion. Another example revealed that SACC-derived exosomes promoted tumor metastasis by educating human periodontal ligament fibroblasts regarding the protumorigenic phenotype.^92^

On the other hand, CAF-derived EVs can alter tumor cells gene expression.^93^ Principe et al.^94^ identified MFAP5-enriched CAF-derived EVs as a key player in oral tongue squamous cell carcinoma progression. CAF-derived EVs could promote lymph node metastases in OSCC by encapsulating ITGB1 and BMI1.^95^ Besides proteins, researchers confirmed that CAFs promoted OSCC proliferation and metastasis via downregulated exosomal miR-34a-5p,^96^ whereas overexpressed CAFs-exosomal miR-382-5p^97^ and miR-146b-5p^98^ improved OSCC proliferation, migration, and invasion. The loss of CAF-exosomal miR-3188 could also promote HNSCC progression via the derepression of BCL2.^99^ Moreover, CAF-derived EVs conferred cisplatin resistance in HNSCC via miR-876-3p^100^ and miR-196a.^101^

### EVs influence drug resistance

Cisplatin resistance is a primary obstacle to effective OSCC treatment. Exosomes confer cisplatin resistance via miRNAs transference^102,103^ (Fig. 5e). Besides cisplatin resistance, EVs secreted by clinically radioresistant OSCC cells facilitated the acquisition of radioresistance via the miR-503-3p-BAK axis.^104^ Nonetheless, EVs may also serve as a target for cancer therapy. Inhibition of tumor cell-derived EVs secretion increased drug sensitivity in cisplatin-resistant H314 cells.^105^ Kalia K and his colleagues^106,107^ discovered that exosomal miR-155 conferred cisplatin chemoresistance in OSCC cells by promoting epithelial-mesenchymal transition; this process was reversed by exosomes-loaded with the miR-155 inhibitor. Moreover, exosomes from the cisplatin-resistant OSCC cells regained cisplatin sensitivity after transfection with miR-30a mimics.^108^

## Stem cell-derived EVs

Numerous studies have highlighted the potential of stem cells in tissue engineering, immunotherapy, and regenerative medicine owing to their capacities for self-renewal, multi-directional differentiation, and immunoregulation.^109^ Interestingly, prevailing evidence indicates that many of the positive effects of stem cell-based therapy are due to their paracrine effects rather than cell replacement.^110,111^ As a critical component of paracrine activity, EVs derived from oral tissue stem cells exhibit specific biological characteristics and functions, particularly inducing tissue regeneration, immunomodulation, and promoting angiogenesis.^5,112–115^ Here, we will focus on the functional mechanisms of stem cell-derived EVs in tissue regeneration.

### Craniomaxillofacial bone defects

**Fig. 6:**
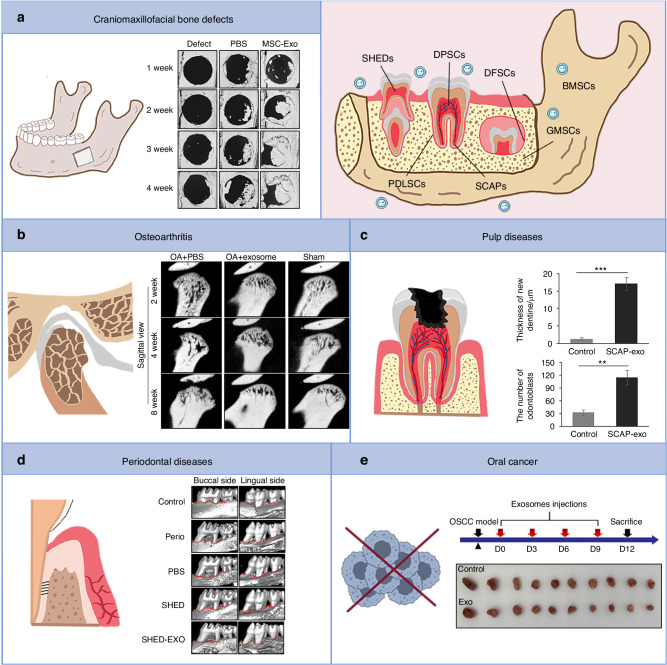
Biological function of EVs derived from oral tissue stem cells. **a** EVs promote bone regeneration in craniomaxillofacial defects.^125^ **b** EVs have therapeutic effects on OA.^133^ **c** EVs mainly DPSC-EVs promote pulp regeneration and repair.^147^ **d** EVs promote both periodontal bone and soft tissue regeneration and ameliorate periodontitis.^168^ **e** EVs exerting an anti-tumor effect on tumor development.^182^ BMSCs bone marrow mesenchymal stem cells, DPSCs dental pulp stem cells, SHEDs stem cells from exfoliated deciduous teeth, PDLSCs periodontal ligament stem cells, DFSCs stem cells from dental follicle, SCAPs stem cells from apical papilla, GMSCs gingival mesenchymal stem cells, OA osteoarthritis

Bone defects arising from trauma, tumors, and surgeries are a significant issue in craniomaxillofacial surgery. Research has indicated that MSC-EVs can induce osteogenesis, immune modulation, and angiogenesis, thereby facilitating bone regeneration (Fig. 6a). MSC-derived EVs may be a potential cell-free therapy for craniofacial bone repair.

MSC-EVs can regulate the osteogenic differentiation of various stem cells. DPSC-EVs have been shown to promote the osteogenic differentiation capability of jawbone marrow-derived MSCs.^116^ Another report indicated that DPSC-EVs promoted osteogenic differentiation of adipose-derived mesenchymal stem cells (ADSCs) via the MAPK signaling pathway.^117^ Guo et al.^118^ isolated exosomes from stem cells from exfoliated deciduous teeth (SHEDs), and found that they enhanced the osteogenic differentiation of DPSCs. Additionally, ABs released by apoptotic BMSCs could efficiently promote the osteogenic differentiation of recipient BMSCs and improve craniofacial bone repair.^119^

The immune microenvironment is a key regulator of bone regeneration, and stem cell-derived EVs have been reported to promote bone regeneration by exerting immunomodulatory effects. Wei et al.^120^ suggested that exosomes extracted from various stages of osteogenically differentiating BMSCs had inflammation-regulatory capacities on macrophages. Elsewhere, another study suggested that EVs derived from TNF-αpreconditioned BMSCs could promote bone regeneration indirectly by changing macrophage phenotype.^121^ In a study conducted by Li et al.^122^ it was observed that ADSC-exosomes could effectively regulate the immune metabolism of skeletal tissue by promoting a shift in macrophage phenotype towards M2, which further enhanced bone healing in the repair of craniomaxillofacial bone defects. Additionally, BMSC exosomes could mitigate radiation-induced bone loss by reinstating the function of recipient BMSCs.^123^ In inflammatory jawbone defects caused by periapical periodontitis, umbilical cord mesenchymal stem cell (UCMSC)-EVs were found to enhance the proliferation and migration of BMSCs, outperforming traditional bone meal treatment.^124^

Stem cell-derived exosomes could promote bone regeneration by improving angiogenesis.^125,126^ The hypoxic microenvironment has been demonstrated to promote the proangiogenic activity of MSCs. sEVs from hypoxia-preconditioned MSCs could promote HUVECs angiogenesis, ultimately contributing to vascularized bone regeneration in a critical-size calvarial bone defect model.^127^ Similarly, exosomes derived from BMSCs preconditioned under hypoxic conditions exhibited enhanced proangiogenic effects in calvarial bone regeneration.^128^

### Osteoarthritis

Osteoarthritis (OA) is a degenerative disease that causes chronic pain and functional limitations with no effective treatment available. Studies indicate that stem cell-derived EVs have a therapeutic effect on OA (Fig. 6b). BMSC-EVs promoted human cartilage regeneration in vitro,^129^ whereas BMSC exosomes could also ameliorate OA by inhibiting pyroptosis of chondrocytes and cartilage.^130^ It has been demonstrated that DPSC-exosomes can protest against OA development. Intra-articular injection of DPSC-exosomes was reported to alleviate abnormal subchondral bone remodeling, improve cartilage matrix degradation, and inhibit synovial inflammation of knee OA.^131^ Exosomes derived from DPSCs overexpressing miR-140-5p were found to decrease OA symptoms by inhibiting chondrocyte apoptosis and promoting cartilage repair.^132^ However, research on temporomandibular joint (TMJ)-OA is relatively limited compared with peripheral joint OA currently. For the first time, Zhang et al.^133^ explored the role of MSC-exosomes in TMJ OA and noted that they could promote TMJ repair and regeneration by suppressing inflammation and restoring matrix homeostasis. In another study, BMSC-sEVs induced cartilage reconstruction in chondrocytes from TMJ OA via the autotaxin-YAP signaling axis in chondrocytes.^134^ In addition, SHED-exosomes significantly suppressed inflammation in TMJ chondrocytes via miR-100-5p/mTOR in vitro.^135^ Researchers developed a rat model of bisphosphonate-related osteonecrosis of the jaws and found that MSC-EVs could mitigate zoledronic acid-induced senescence of stem cells, osteoblasts, and fibroblasts, and decrease the production of inflammatory cytokines, thereby preventing the disease.^136^

### Pulp diseases

Pulp disease treatment aims to promote the regeneration of functional pulp tissue. Although pulp revascularization and tissue engineering have shown promising clinical results, their limitations have hindered their widespread use.^137,138^ Stem cell-derived EVs have shown great potential to enhance pulp regeneration as cell-free therapies^139^ (Fig. 6c). The property of EVs to accelerate pulp regeneration primarily originates from DPSCs.

The immune microenvironment plays a crucial role in pulpitis treatment. In an LPS-induced mild inflammatory microenvironment, DPSC-sEVs made the regenerated pulp more similar to the normal structure by modulating BMSCs.^140^ Additionally, DPSC-exosomes enhanced odontogenesis by polarizing macrophages towards the pro-healing M2 phenotype.^141^ Yu et al.^142^ discovered that exosomes derived from stem cells from apical papilla (SCAPs) could promote Treg conversion and effectively inhibit rat dental pulp inflammation.

EVs can promote pulp regeneration by directly regulating MSCs proliferation, migration, and odontogenic differentiation. In 2016, Huang et al.^17^ first demonstrated the feasibility of using exosomes for pulp regeneration. They isolated exosomes from DPSCs under odontogenic conditions and found that DPSC-exosomes significantly stimulated DPSCs odontogenic differentiation and triggered dental pulp-like tissue formation in vivo. Moreover, DPSC-exosomes promoted the migration and osteogenic differentiation of MSCs, thereby enhancing dental pulp restoration.^143^ Schwann cells are important sources of dental MSCs, which can move to the injured sites and differentiate into odontoblasts and pulp cells.^144^ Exosomes derived from Schwann cells promoted DPSCs proliferation and multipotency,^145^ while LPS-preconditioned DPSC-exosomes enhanced Schwann cell odontogenic differentiation in vitro.^146^ The positive feedback loop between Schwann cells and DPSCs, mediated by exosomes, further amplifies the functional capabilities of DPSCs. Beyond DPSC-exosomes, SCAP-exosmes significantly enhanced the dentinogenic potential of BMSCs and stimulated the formation of new dentin and pulp-like tissues within BMSC-containing root fragments.^147^

Pulp vascularization is essential for successful pulp regeneration. Huang et al.^148^ revealed for the first time that LPS-stimulated DPSC-exosomes possessed better proangiogenic potential in vitro. Li et al.^149^ further verified that hypoxia augmented the angiogenic potential of DPSC-derived exosomes and partially altered their proteome profile. Researchers discovered that ABs derived from hDPSCs could stimulate the growth of host blood vessels in an ischemic hypoxic environment, ultimately promoting pulp regeneration.^150^ In addition, SHED aggregate-derived exosomes not only promoted SHED endothelial differentiation but also enhanced HUVECs angiogenic potential, contributing to angiogenesis in pulp tissue regeneration.^151^

Pulp nerves repair represents a significant challenge in pulp regeneration. Compelling evidence has shown that exosomes derived from dental stem cells facilitate peripheral nerve regeneration.^152–155^ Despite their potential benefits, the exact functions and mechanisms through which exosomes directly promote dental pulp nerve regeneration remain elusive.

### Periodontal diseases

Periodontitis is a highly prevalent chronic inflammatory disease characterized by the breakdown of periodontal tissues and eventual tooth loss. Excessive host immune response predominantly promotes tissue damage and disease progression in this condition. Nonetheless, many studies have focused on the therapeutic applications of EVs in periodontitis rather than their pathogenic effects, providing evidence of stem cell EVs as a cell-free strategy for periodontal regeneration (Fig. 6d). In comparison to the primary application of DPSC-EVs in pulp regeneration, various stem cell-derived EVs are involved in periodontal regeneration.

Numerous studies demonstrated that stem cell-derived EVs regulate immune cell function and ameliorate periodontal inflammation. BMSC exosomes ameliorated periodontitis since they could suppress inflammatory response induced by *P. gingivalis* in macrophages in vitro, as well as reduced tissue damage and immune cell infiltration in vivo.^156^ Both BMSC-sEVs and BMSC-ABs could inhibit the periodontitis progression and tissue damage by modulating osteoclast function and macrophage polarization.^157,158^ It was previously reported that hBMSC-EVs restored Th17/Treg homeostasis in periodontitis via miR-1246.^159^ These findings demonstrate a promising therapeutic potential of BMSC-EVs in the treatment of periodontitis. Gingival mesenchymal stem cell (GMSC)-exosomes could reduce the pro-inflammatory factors produced by M1 macrophages and induce a switch to the anti-inflammatory M2 phenotype,^160^ whereas exosomes from TNF-α-treated GMSCs improved M2 macrophage polarization and prevented periodontal bone loss.^161^ Moreover, GMSC-exosomes were capable of promoting the polarization of LPS/INF-γ-induced inflammatory macrophages into anti-inflammatory phenotype.^162^ Another study found that GMSC-exosomes suppressed the inflammatory response of periodontal ligament stem cells (PDLSCs).^163^ Besides, Han et al.^164^ discovered that PDLSC-EVs delivered miR-590-3p into macrophages, inhibiting TLR4 transcription, subsequently reducing macrophage pyroptosis, and attenuating periodontal inflammatory damage. Exosomes from PDLSCs in an LPS-induced periodontitis environment could modulate Th17/Treg balance and alleviate inflammatory microenvironment.^165^

Alveolar bone regeneration is the most extensively studied area within periodontal regeneration. Similar to the craniomaxillofacial bone repair discussed previously, periodontal bone regeneration is also characterized by unique challenges posed by inflammation and microorganisms. Stem cell-derived EVs can promote periodontal bone regeneration by modulating osteogenic differentiation, angiogenesis, as well as osteoblast and osteoclast activity. Wang et al.^166,167^ revealed that SHED-exosomes significantly improved hPDLC osteogenic differentiation. In another way, SHED-exosomes directly promoted BMSC osteogenesis and bone formation.^168^ Moreover, SHED-EVs promoted the repair of alveolar bone defect by targeting HUVECs angiogenesis and BMSCs osteogenesis.^169^ Elsewhere, DPSC-exosomes regulated the anti-inflammatory and osteogenic properties of PDLSCs, induced a phenotypic shift in macrophages from M1 to M2, and promoted the healing of alveolar bone and periodontal epithelium in rats with experimental periodontitis.^170^ PDLSCs exhibit a greater osteogenic potential than DPSCs, while displaying distinctive capabilities for generating periodontal tissues, making it an ideal candidate for the treatment of periodontal bone loss.^171,172^ Xie et al.^173^ found significant alterations in the expression of urinary circRNAs and lncRNAs in PDLSC-exosomes during the early stages of osteogenic differentiation of PDLSCs. In addition, PDLSC-exosomes were capable of promoting proliferation, migration, and anti-apoptotic effect of osteoblasts.^174^ LPS-preconditioned dental follicle cell-derived sEVs could promote the proliferation, migration, and differentiation of periodontal ligament cells from periodontitis in vitro and in vivo.^175^ A recent study indicated that BMSC exosomes could promote PDLSCs osteogenic differentiation via lncRNA HCP5.^176^ Besides, researchers isolated ABs from BMSCs and found that these ABs could suppress osteoclast differentiation by transferring miR-223-3p, leading to a significant reduction in bone loss in an animal model of periodontitis.^177^

The process of periodontal regeneration typically involves the remodeling of periodontal soft tissues, such as the gingiva. Using a palatal gingival defect model in rats, Liu et al.^178^ found that SCAP-exosomes could improve angiogenesis and effectively promote gingiva regeneration. Besides, Kou et al.^179^ uncovered that GMSC-sEVs containing IL-1RA promoted gingival wound healing.

### Oral cancer

MSC-exosomes have been reported to modulate the TME. For instance, BMSC exosomes could transfer miR-101-3p to OSCC cells, leading to the downregulation of COL10A1 and subsequent inhibition of OSCC growth.^180^ Rosenberger et al.^181^ employed hamster buccal pouch carcinoma as a preclinical model mimicking human OSCC, and found that stem cell-derived exosomes could suppress angiogenesis and tumor growth. In a separate study, Liu et al.^182^ established an OSCC xenograft transplantation model and observed that SHED-exosomes inhibited tumor microvascular formation and exerted an anti-tumor effect (Fig. 6e).

In addition to their anti-tumor properties, EVs can facilitate the repair of postoperative tumor defects, including bone defect regeneration discussed above, as well as soft tissue repair. Although there are numerous articles investigating the function of diverse MSC-derived EVs in wound healing and tendon and skeletal muscle repair,^183–191^ maxillofacial skin and muscle regeneration remains relatively understudied. Tongue reconstruction is a critical component of post-treatment care for tongue cancer. Although flap grafts remain the primary approach, regenerative medicine using stem cells has been explored in recent years to restore tongue function and taste sensation. Zhang et al.^192^ examined the role of exosomes in this process and discovered that a combination of exosomes derived from GMSCs and a small intestinal submucosa-extracellular matrix structure could promote taste bud regeneration and reinnervation.

## Body fluid-derived EVs

**Fig. 7:**
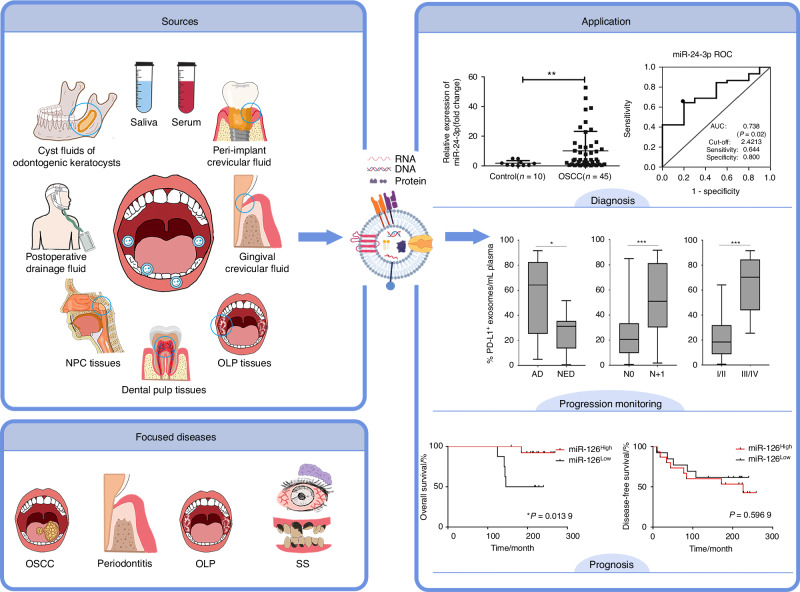
EVs derived from body fluids and tissues in the oral and maxillofacial region. EVs have been detected in various sources within the oral and maxillofacial region, including various body fluids and tissues. Current researchers are investigating the roles of EVs in diseases including OSCC, periodontitis, OLP, and SS. Differentially expressed cargos in EVs offer a promising strategy for disease diagnosis, progression monitoring, and prognosis prediction.^195,203,221^ NPC nasopharyngeal cancer, OSCC oral squamous cell carcinoma, OLP oral lichen planus, SS Sjögren’s syndrome

Considering that EVs can mirror the altered physiological and pathological condition of their parent cells, increasing attention has focused on EVs derived from diverse biological fluids, such as blood, saliva, breast milk, cerebrospinal fluid, and urine (Fig. 7). Whiteside TL and his colleagues^193,194^ first isolated tumor-derived microvesicles from plasma of patients with HNSCC; they found that these FasL-positive microvesicles could improve immune suppression and apoptosis of activated immune cells. Studies suggest that body fluid-derived EVs hold significant promise as novel biomarkers in liquid biopsy and reflect the dynamic progression of diseases in real time, thereby providing a minimally invasive strategy for disease diagnosis, progression monitoring, and prognosis prediction. Exosome diagnostic applications have been targeted on diseases including periodontitis, OLP, and OSCC.

The diagnostic potential of salivary exosomal miRNAs as biomarkers for OSCC screening has been reported. He et al.^195^ analyzed salivary EVs from OSCC patients and healthy controls via miRNA microarray analysis; they found that miR-24-3p had outstanding diagnostic accuracy for OSCC. Similarly, Gai et al.^196^ discovered a subset of miRNAs specifically enriched in salivary EVs of OSCC patients whereas Patel et al.^197^ identified three downregulated salivary exosomal miRNAs for predicting OSCC progression and poor prognosis. A previous systematic review uncovered four miRNAs in salivary EVs with high potential to be biomarkers of HNSCC diagnosis.^198^ He et al.^199^ evaluated serum EVs of 184 OSCC patients and 196 healthy individuals and found considerably higher miR-130a levels in OSCC patients. Besides miRNAs, differentially expressed proteins in serum EVs can facilitate early detection of metastasis on OSCC.^200,201^ Serum/salivary Alix levels in EVs are significantly higher in OSCC patients.^202^ The serum EVs PD-L1 levels in HNC patients are associated with disease activity and progression.^203,204^ Feng et al.^205^ examined EV phenotypes via a microfluidic approach and found that OSCC patients had higher levels of EGFR^+^ salivary EVs compared to healthy individuals, and the ratio of Annexin V^+^ EGFR^+^ EVs to Annexin V^-^ EGFR^+^ EVs was negatively correlated with tumor T stage. In addition, Wang et al.^206^ compared the proteomic profile of EVs from postoperative drainage fluid in patients with metastatic and nonmetastatic OSCC and identified differentially expressed proteins. Additionally, cyst fluids of odontogenic keratocysts contain significantly higher levels of cell-derived microparticles (MPs) compared to dentigerous cysts and radicular cysts, which are closely correlated with cyst diameters.^207^

Detection of salivary and serum EVs could be a vital non-invasive method in diagnosing and evaluating the state of periodontitis and peri-implantitis. Differentially expressed miRNAs are detected in serum and salivary exosomes compared to healthy samples, which may be promising biomarkers for periodontitis.^15,208,209^ Patients with periodontitis could be differentiated from both healthy controls and gingivitis patients by the global 5mC hypermethylation in salivary sEVs.^210^ Yu et al.^211^ identified higher levels of PD-L1 mRNA in salivary exosomes of periodontitis patients, correlating with the stages of periodontitis. As for proteins, CD9 and CD81 levels are significantly decreased in salivary exosomes of periodontitis patients, and are negatively correlated with the stage and grade of the disease.^212^ Kwon et al.^213^ profiled serum-derived exosomal RNA and discovered that specific miRNAs were downregulated during periodontitis and recovered to healthy levels after treatment. Gingival crevicular fluid is a promising oral biofluid that can aid in differentiating between periodontal health and disease status, with studies showing increased total gingival crevicular fluid-EVs concentrations in periodontitis patients compared to healthy/gingivitis subjects.^214,215^ Chaparro et al.^216^ first isolated and identified the presence of EVs in the peri-implant crevicular fluid. They found a significant increase in the concentration of peri-implant crevicular fluid-EVs, microvesicles, and exosomes in peri-implantitis implants, as well as a correlation between miRNAs downregulation and peri-implantitis.

The application of EVs in SS and OLP diagnosis has also been documented. Michael et al.^217^ first attempted to isolate and characterize salivary exosomal miRNAs from SS patients. Li et al.^218^ further identified two upregulated circRNAs in plasma exosomes from SS patients, whereas two upregulated miRNAs were found in serum exosomes of an SS mouse model in another study.^219^ Significantly upregulated miR-4484 in the salivary exosomes of OLP patients was proposed to be a biomarker for OLP.^14^

In addition to their diagnostic potential, exosomes can help in disease prognosis. Patel et al.^220^ found that the enhanced expression of miR-1307-5p in salivary exosomes was clinically associated with poor patient survival in OSCC. Besides, substantially decreased levels of serum exosomal miR-126 were detected in the late-staged OSCC patients, which might be of significance in poor survival.^221^ Similarly, OSCC patients with high expression of serum exosomal miR-130a exhibited worse 3-year overall survival and recurrence-free survival.^199^

## Tissue-derived EVs

Compared to EVs obtained from cell lines and body fluids, tissue-derived EVs (Ti-EVs) exhibit tissue specificity and yield a more precise depiction of the actual physiological or pathological condition of the tissue microenvironment.^222^ Additionally, Ti-EV samples exhibit minimal contaminants due to their single-tissue source in contrast to body fluid‐derived EVs.^223^ Sun et al.^224^ extracted Ti-EVs from OLP and oral lichenoid lesions and noted that the upregulation of protein disulfide isomerase family A member 3 in EVs could potentially contribute to the development of both diseases. Exosomes from dental pulp tissues improved odontogenic and neurogenetic differentiation of SCAPs and revascularization, as well as performed better in pulp regeneration unlike DPSC-EVs^225^ (Fig. 7).

Nonetheless, studies on Ti-EVs in the oral and maxillofacial region are limited. Improving comprehension of the intrinsic properties of Ti-EVs and investigating better-standardized protocols for their characterization will broaden the potential for their clinical implementation.

## Engineered exosomes in the dental and craniofacial field

Because of their structure and characteristics, including high biocompatibility, minimal toxicity, and immunogenicity, the capacity to encapsulate endogenous physiologically active molecules and cross the blood-brain barrier, exosomes can serve as natural nanocarriers for therapeutic agents and drugs. Nevertheless, the eventual clinical translation of exosomes is still with numerous challenges including large-scale manufacture and quality control. Exosomes can be called “programmable exosomes” or “engineered exosomes” after synthesis or modification by various biotechnologies. Engineering exosomes using nanobiotechnology may resolve the challenges of natural exosomes since they have great clinical application potential in various fields including tumor, tissue regeneration, and repair; they have also demonstrated better therapeutic effect and targeting capacity compared to natural exosomes. This work summarizes the several potential approaches to engineering exosomes.

### Artificial exosomes

**Fig. 8:**
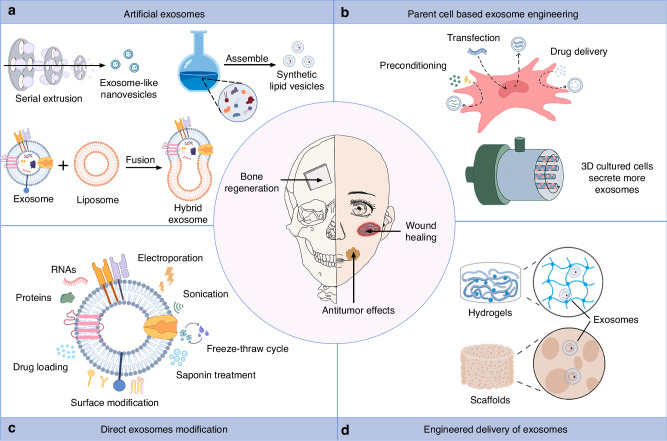
Engineered exosomes in craniofacial and dental fields. **a** Artificial exosomes can be developed through nanobiotechnology, which consists of top-down strategies, bottom-up strategies, and biohybrid strategies. **b** Parent cells can be modified in various ways to give exosomes therapeutic properties. 3D culture improves exosome yields. **c** Exosomes can be modified by altering their surface proteins or contents. **d** Combining exosomes with hydrogels and scaffolds is a better way to deliver exosomes

Regarding the limitations of natural exosomes, three major strategies, top-down, bottom-up, and biohybrid, have been utilized to develop artificial exosomes^226^ (Fig. 8a and Supplementary Table 2).

#### Biomimetic vesicles

In top-down strategies, parent cells are processed through microfluidic and extrusion technologies to obtain small membrane fragments, which are self-assembled into exosome-like nanovesicles. Jang et al.^227^ first developed bioinspired exosome-mimetic nanovesicles by extruding cells through filters with progressively smaller pore sizes. These nanovesicles are not only 100 times more productive but also have similar properties to exosomes, as well as the natural targeting capacity of cells. Similarly, Zhu et al.^228^ developed NK cell-derived exosome mimetics using filters with decreasing pore sizes, exerting natural targeting capacity and strong killing effects on cancer cells. Yoon et al.^229^ proposed a microfluidic system that produced nanovesicles of 100–300 nm by slicing living cell membranes with microfabricated 500 nm-thick silicon nitride blades. Ultrasound can be used to assemble fragments of cell membranes into small vesicles. Go et al.^230^ developed dexamethasone-loaded nanovesicles through a process involving alkaline solution, sonication, and density gradient ultracentrifugation, which had a 200-fold higher yield than EVs. Additionally, Wang et al.^231^ utilized ultrasonication to generate nanoscale-engineered EVs which had ~20-fold higher yield and ~100-fold faster production.

In bottom-up strategies, small molecules are assembled to form nanovesicles, with higher stability but limited biocompatibility.^232^ Vázquez-Ríos et al.^233^ developed exosome-mimicking nanosystems that not only mimicked the structure of natural tumor-derived exosomes but also facilitated targeted drug delivery. The binding of APO2L/TRAIL to liposomes resulted in the production of artificial lipid vesicles, which demonstrated therapeutic potential in the treatment of rheumatoid arthritis and hematological tumor.^234–236^ Furthermore, Zheng et al.^237^ fabricated triptolide-loaded membrane protein-chimeric liposomes, and demonstrated that they exhibited good tumor targeting while mitigating the toxicity of triptolide. Yu et al.^238^ conjugated a CD11c monoclonal antibody to the surface of immunoliposomes to achieve Langerhans cells targeted. They found that this strategy improved the efficiency of antigen presentation and activated cellular immune responses in vivo.

#### Hybrid exosomes

Hybrid exosomes are formed by merging synthetic nanoparticles and natural exosomes using biohybrid techniques, the most common of which is exosome and liposome fusion. Sato et al.^239^ fused the exosomal membranes with liposomes by freeze-thaw techniques, yielding hybrid exosomes with a higher cellular uptake rate. Lin et al.^240^ fabricated exosome-liposome hybrid nanoparticles to deliver CRISPR-Cas9 expression vectors in MSCs. In addition, Piffoux et al.^241^ revealed that polyethylene glycol could cause fusion between EVs from different cellular sources and functionalized liposomes; the resulting hybrid EVs showed efficient drug loading and delivery. Rayamajhi et al.^242^ endowed the hybrid exosomes with both the endogenous nature of EVs and the flexibility of liposomes using extrusion-based membrane fusion technique, making it a promising tumor-targeted drug delivery candidate. Using similar techniques, Hu et al.^243^ fused C-X-C motif chemokine receptor 4-positive exosomes with liposomes transporting antagomir-188, which significantly reversed age-related bone loss by promoting osteogenesis of BMSCs and suppressing adipogenesis.

### Exosomes modification

#### Indirect modification-parent cell-based engineering

Engineered exosomes can be produced by modifying parental cells through various methods, including genetic engineering, co-incubation, and altered cell culture conditions (Fig. 8b and Supplementary Table 3).

In the conventional approach, cells are transfected with recombinant viruses or plasmids to generate exosomes carrying specific genes. For instance, Altanerova et al.^244^ treat MSCs with recombinant retrovirus carrying the yCD::UPRT fused gene to secret exosomes encoding the suicide gene, which subsequently induced dose-dependent tumor cell death. Two studies demonstrated that decreased levels of LncRNA ADAMTS9-AS2 and LncRNA PART1 were correlated with worse outcomes in OSCC patients.^245,246^ The transfection of OSCC cells with these two lncRNAs via lentiviral vectors resulted in inhibition of the malignant progression of OSCC. A previous study found that hASCs transfected with lentiviral vectors produced exosomes carrying therapeutic miR-375, which enhanced bone regeneration in rat calvarial defect.^247^ Another study genetically modified MSCs with bone morphogenetic protein 2 lentivirus and generated MSC-EVs with enhanced bone regeneration ability in a rat model of calvarial defects.^248^ Luo et al.^249^ developed C-X-C motif chemokine receptor 4-overexpressing exosomes-loaded with miR-126 through lentiviral vector and miRNA transfection, which mitigated periodontitis and prevented alveolar bone loss. Nuclear factor I/C (NFIC) is a key transcription factor in tooth root development. Yang et al.^250^ constructed NFIC-encapsulated EVs by overexpressing NFIC in HEK293FT cells, which promoted dentin formation. Engineered exosomes can also promote wound healing.^251^ HEK293 cells transfected with lentivirus vectors were used to produce engineered miR-31 exosomes as a RNAi therapeutic agent to promote diabetic wound healing.^252^ Plasmids were utilized to transfect MSCs to produce EVs loaded with HOX transcript antisense lncRNA.^253^ It was observed that they promoted angiogenesis and wound healing in diabetic mice.

Moreover, co-culture with parent cells allows for the loading of drugs onto exosomes. Pascucci et al.^254^ incubated Paclitaxel with MSCs and found that the isolated exosomes had potent anti-proliferative activity against pancreatic cancer, demonstrating for the first time that MSCs could package and deliver active drugs via their exosomes.

Treating cells with physical materials, such as magnetic nanoparticles and static magnetic fields can prepare nucleic acid-bearing exosomes. Labeling yCD::UPRT-expressing MSCs with Venofer, an iron oxide carbohydrate nanoparticle, could produce exosomes packaged with magnetic nanoparticles for targeted tumor cell ablation via magnetically induced hyperthermia.^255^ Highly enriched miR-21-5p was detected in exosomes released from BMSCs preconditioned with Fe_3_O_4_ nanoparticles and a static magnetic field, and was found to improve wound healing.^256^

Optimizing cell culture conditions presents a strategy to not only enhance EVs production but also modulate their functionality and properties. Studies have demonstrated a significant 7.5-fold increase in the yield of exosomes derived from UCMSCs cultured in three-dimensional (3D) cultures compared to 2D cultures.^257^ Moreover, these 3D-derived exosomes exhibited superior osteochondral regenerative activity. Similarly, Yu et al.^258^ found that 3D exosomes promoted the osteogenic gene and protein expression of hBMSCs, and accelerated new bone formation in a rat alveolar bone defect model. The 3D culture system improved the therapeutic effects of MSC-exosomes in periodontitis and experimental colitis.^259^ Further, by combining tangential flow filtration with scalable microcarrier-based 3D cultures, the yield of MSC-exosomes was sevenfold higher compared to 3D exosomes.^260^ Duan et al.^261^ developed a novel method to produce high-purity artificial EVs from DPSC lysates. These artificial EVs exhibited similar properties to native EVs, but could be extracted 16 times more efficiently. Other methods, including molecular interference, environmental factors, and external inducers have also been investigated to elevate EV secretion rates by up to two orders of magnitude.^262^

#### Direct exosomes modification

Several strategies have been developed to directly prepare engineered exosomes, including co-incubation, physical methods, saponin, and surface modification, all of which modify the surface proteins or contents of purified exosomes (Fig. 8c and Supplementary Table 4).

Co-incubation of cargos with isolated exosomes is a simple and common method used for loading therapeutic agents into exosomes. For example, co-incubation successfully loaded cholesterol-modified miR-34a into HEK293T cell-derived exosomes, significantly inhibiting proliferation, migration, and invasion of OSCC.^263^ Additionally, Jang et al.^264^ designed an engineered EV exogenously loaded with cyclic dinucleotide, termed exoSTING, to promote tumor immune surveillance. However, these approaches are limited by low drug-loading efficiency, particularly for hydrophobic molecules.

Physical methods such as sonication, electroporation, and freeze-thaw allow researchers to prepare therapeutic agents that enter exosomes by destroying their membrane integrity. Cutler CW and colleagues^265,266^ employed sonication to load TGFB1 and IL-10 into DC exosomes, and subsequently demonstrated the ability of these engineered exosomes to modulate human DCs and T cells, leading to the inhibition of bone loss in periodontitis. Cui et al.^267^ fabricated melatonin-loaded engineered M2 exosomes via ultracentrifugation and cyclic sonication, to induce macrophage reprogramming towards M2 and accelerate periodontal regeneration. In terms of tumor treatment, a study found that loading Paclitaxel into M1 exosomes by sonication caused stronger anti-tumor effects than the M1 exosomes or Paclitaxel groups.^268^ To develop OSCC-targeting exosomes, Kase et al.^269^ used siRNA of lymphocyte cytoplasmic protein 1 (LCP1) to electroporate exosomes from normal fibroblasts transfected with EBV Induced-3 cDNA. These exosomes stably and efficiently transferred siLCP1 to inhibit the oncogenic potential of OSCC cells. In addition, hASC-derived exosomes with miR-21-5p mimics loaded via electroporation, exerted strong effects on diabetic wound healing.^270^

### Engineered delivery of exosomes

The utilization of EVs as a cell-free therapeutic modality for tissue repair and regeneration faces certain limitations, primarily concerning the attainment of sustained, localized release in the presence of body fluid dynamics and rapid clearance by the circulatory system. Nonetheless, researchers have identified a promising avenue to address this challenge by integrating EVs with biomaterials characterized by both biocompatibility and biodegradability. This strategy has shown potential to overcome the aforementioned challenges and enhance the efficacy of EV-based therapies^271^ (Fig. 8d).

Biomedical hydrogels, such as hyaluronic acid, chitosan, and polyethylene glycol, are considered to be good carriers for loading and releasing EVs at the site of injury. For instance, Zhang et al.^272^ encapsulated UCMSC-exosomes in hyaluronic acid hydrogel and combined them with customized nanohydroxyapatite/poly-ε-caprolactone scaffolds to repair large cranial defects. Similarly, a study by Wu et al.^273^ developed injectable thermosensitive hydrogel-encapsulated BMSC-sEVs, which significantly extended the delivery and release duration, as well as enhanced bone regeneration and repair of calvarial defects in rats. Li et al.^274^ combined exosomes derived from hADSCs with poly(lactic-co-glycolic acid) scaffolds, these slowly and consistently released exosomes significantly enhanced the restoration of critical-sized calvarial defects. Swanson et al.^275^ demonstrated that triblock copolymer microspheres immobilized on a nanofibrous PLLA scaffold achieved controlled delivery of hDPSC-exosomes to treat mouse calvarial bone defect. In addition, Trubiani O and his colleagues^276,277^ found that in rats with cortical calvaria bone defects, the combination of polyethyleneimine-engineered EVs and 3D-engineered scaffolds provided satisfactory bone healing and regeneration. Zarubova et al.^278^ immobilized GMSC-EVs on the surface of the MPs via an MMP-2 sensitive linker, which achieved the targeted release of the EVs at the site of inflammation. One-time administration of GMSC-EVs-decorated MPs significantly improved periodontal tissue regeneration in a rat model of periodontitis. In an immunocompetent rat periodontal defect model, Chew et al.^279^ found that BMSC exosomes-loaded collagen sponge improved periodontal regeneration by promoting the formation of new bone and periodontal ligament. Another study incorporated DPSC-exosomes with chitosan hydrogel and found that this composition accelerated the periodontal epithelium and alveolar bone repair in periodontitis mice.^280^ Besides, the combination of GMSC-exosomes with chitosan/silk-based hydrogel sponge successfully promoted diabetic skin wound healing.^281^

The combination of biomaterials and EVs carriers may be a promising strategy to prolong EVs retention, meanwhile, EVs can enhance the biomaterials bioactivity. Zhang et al.^282^ found that the hiPSC-MSC-exosomes/tricalcium phosphate combination scaffolds were effective in promoting osteogenesis and repairing calvarial bone defects compared with pure tricalcium phosphate scaffolds. In a rat model of cranial defects, incorporating BMSC-EVs into hierarchical mesoporous bioactive glass scaffolds significantly enhanced the bone-forming capacity of the scaffolds and accelerated bone regeneration.^283^ According to Gandolfi et al.^284^ enrichment of mineral-doped PLA-based porous scaffolds with exosomes increased the osteogenic commitment of hADSCs.

Further investigation to develop better biomaterial-EV systems is warranted.^285^ Moreover, artificial intelligence and 3D printing can potentially optimize the design and fabrication of hydrogels for more personalized clinical applications.^286^

### Clinical application

Exosomes have been demonstrated to have diverse clinical applications in cancer, neurological diseases, cardiovascular diseases, immune diseases, infectious diseases, and tissue regeneration and repair. Their key clinical uses are as biomarkers, cell-free therapeutics, drug delivery vehicles, and cancer vaccines.^287^ ExoASO-STAT6 is an engineered exosome with therapeutic effects developed by Codiak BioSciences, Inc., which selectively delivers antisense oligonucleotides to reduce STAT6 mRNA expression in TAMs.^288^ A previous study explored the application of exoASO-STAT6 in the oral field and found that it may become a potent monotherapy for OSCC treatment.^289^ In a review investigating the current status of exosome application in clinical settings using the ClinicalTrials.gov (https://clinicaltrials.gov/), database showed that 50% of 116 clinical trials were related to biomarker applications, and 28.44% were associated to exosome therapy.^290^ However, the majority of research focused on cancer biomarkers and SARS-CoV-2 pneumonia treatment, with EV-based clinical translation in the oral and maxillofacial region still lagging behind. Further research is necessary to explore the potential uses of EVs in this area and to promote their clinical application.

## Limitations and prospects

Currently, the application of EVs in the oral and maxillofacial region has attracted increasing attention. In this paper we discuss EVs derived from cells, body fluids, tissues, and engineered exosomes, their strengths and weaknesses, as well as research opportunities are summarized in Table 1.

**Table 1:** Comparison of characteristics of EVs from four different sources

Source of EVs	Cell-derived EVs	Body fluid‐derived EVs	Tissue‐derived EVs	Engineered exosomes
Advantages	High purity	Easy to obtain	Organ-specific high-purity EVs with minor contaminants	Stronger drug loading efficiency
	Single‐cell source	Minimal invasiveness	Better reflect the tissue microenvironment	Better targeting
		Indicate the dynamic progression of diseases in real-time	Accurate reflection of pathophysiological status	Effective resistance to body clearance
Disadvantages	Absence of local tissue microenvironment	Insufficient purity: mixture of EVs from other cellular and organ origins	Lack of purity: mixture of EVs from multiple cell populations	Difficulty in large-scale manufacture
	Unable to accurately depict the disease’s dynamic progression	Low concentrations of EVs in the circulatory system	Invasiveness	Insertion of exogenous molecules may affect the biological properties of exosomes
	Decreased disease feature representativeness following prolonged cell culture		Restricted sources of sampling	
Applications	Mechanism study	Mechanism study	Mechanism study	
		Diagnostics and prognostics	Potential diagnostics and prognostics	
	Therapeutics	Therapeutics	Potential therapeutics	Therapeutics

*EVs* extracellular vesicles

Despite the inherent limitations of EVs derived from various sources, the clinical application of EVs in oral science is confronted with numerous practical technical obstacles. The major challenge in EVs research lies in the absence of standardized isolation protocols that guarantee both high yield and purity. Unlike synthetic nanoparticles, EVs are naturally produced by cells, hindering large-scale, controlled production. Besides, their heterogeneity in size, cargo, function, and cellular origin significantly complicates their isolation. Currently, no universally accepted methods exist for isolating and purifying EVs from cells or biofluids. Differential ultracentrifugation remains the primary technique for exosome separation, although other established methods like ultrafiltration, polyethylene glycol precipitation, immunoaffinity capture, and size-exclusion chromatography are also employed.^291,292^ Microfluidics and other novel approaches are also emerging, but each technique has specific limitations. To improve the separation efficiency and enrichment, the combination of different methods is increasingly being adopted to enhance the isolation and purification of exosomes. New advanced methods, ranging from upstream processing for MSC production using various bioreactors to downstream processing, have also been developed to increase MSC cultivation and EV yields.^292^ More integrated, high-purity, high-throughput, high-recovery-rate devices need to be developed. In addition, the optimal EV storage conditions (temperature or pH) have not been determined, and strategies for improving targeting and optimizing EV loading capacity should be further explored. It is also crucial to systematically evaluate the safety of EV local injection and systematic administration before clinical trials. Further research is warranted to address these obstacles and improve the application of EVs in various clinical aspects.

To ensure the quality and effectiveness of exosome-based therapeutics for clinical use, it is crucial to establish standardized production methods. A systematic comparison between EVs and well-established traditional methods is essential to provide more robust evidence for the clinical application of EVs. In the future, focus should be directed to the nanoengineering of exosomes to investigate how drug loading efficiency, cost and preparation time, and biosafety of exosome-based therapeutics can be improved.

In conclusion, EVs derived from different sources exhibit distinctive characteristics and can transport diverse cargos to different recipients, thereby modulating most physiological and pathological processes, as well as displaying a variety of applications, including disease diagnosis, tissue repair, and drug delivery. Cell-free therapies based on engineered exosomes can be leveraged to improve disease treatment. The research of EVs in other fields might also provide some reference for oral and maxillofacial applications. Beyond mammalian cell-derived EVs, microbial EVs and plant-derived EVs make up a significant portion of the EV population and have crucial roles in disease development, diagnosis, and therapeutic applications. EVs offer promising opportunities for future research and investigating the characteristics of EVs from different sources could enhance their potential for diagnosing and treating oral diseases and disorders in other body regions.

## Supplementary information

Supplementary information
