# Supplementary material for: Emerging roles of extracellular vesicles in oral and maxillofacial areas
Source: Int J Oral Sci. 2025 Feb 4;17:11. doi: 10.1038/s41368-024-00341-9 (PMC11791077; doi:10.1038/s41368-024-00341-9)
Supplement: Supplementary file 1 — Supplementary information [file 41368_2024_341_MOESM1_ESM.pdf]

Supplementary Table 1. Summary of the biological functions of EVs from different sources via diverse cargos

Type of EVs		Contents of EVs	Biological effects	References
**Epithelial cell-derived EVs**	Oral epithelial cell-derived EVs	miR-135a	Promote tooth tissue development	[6]
		miR-30a, miR-200 family members	Promote EBV reactivation in B cells and EV71 replication during virus infection	[22-24]
	Hertwig's epithelial root sheath cell-derived EVs	/	Promote the regeneration of dentin-pulp tissue	[19]
	Oral mucosal epithelial cells sheets-derived EVs	/	Promote wound healing	[20]
	SGEC-derived EVs	autoantigens Ro/SSA, La/SSB, Sm	Regulate immunologic reactions	[21]
**Immune cell-derived EVs**	Macrophage-derived EVs	miR-378a, IL-10 mRNA, miR-21a-5p, miR-483-5p	Regulate osteogenic differentiation	[35-38,40]
		miR-31-5p, lncRNA LBX1-AS1	Pose a two-sided effect on tumor progression	[45-47]
	B cell-derived EVs	EBV-miR-BART13-3p	Impaire glandular cell function in SS	[28]

	T cell-derived EVs	miR-142-3p	Impaire glandular cell function in SS	29
		interleukin-7, -10, -12, -17, interleukin-1β, -5, interferon-γ	Drive the trafficking of CD8+ T cells and induce keratinocytes apoptosis to promote OLP progression	30-31
	Dendritic cell-derived EVs	SASP	Promote and amplify senescence in normal bystander dendritic cells and T cells	25-27
		MHC II	Suppress inflammation and collagen-induced arthritis	32
		/	Inhibit tumor progression	44
**Tumor-derived EVs**	OSCC cell-derived EVs	miR-142-3p, miR-1246, miR-200c-3p, EGFR, HSP90, miR-21, LOXL2	Promote OSCC cells proliferation, migration and invasion	8,48-54
		miR-210-3p, miR-221, miR-142-3p, miR-130b-3p, miR-23b-3p, EPHB2, adenosine	Promote angiogenesis	10,60-63,69-70
		miR-29a-3p, CMTM6, THBS1, miR-9, PD-L1,	Regulate tumor immunology by reprogramming	9,77-80,82-84

	galectin-1, NAP1	immune cells	
	miR-192/215, TGFβ1	Promote the activation of CAFs	10,88-90
	miR-21, miR-503-3p, miR-155, miR-30a	Enhance OSCC cisplatin resistance and radioresistance	102,104-108
NPC cell-derived EVs	MMP13, EGFR, HMGA2	Promote NPC cells proliferation, migration and invasion	56-59
	miR-205-5p, miR-17-5p, miR-9, miR-144, miR-23a, angiogenic proteins, HAX1, HMGB3	Promote angiogenesis	64-68,71,73,75
	CCL20	Regulate tumor immunology	85
	LMP1	Promote the activation of CAFs	91
	miR-106a-5p	Enhance NPC cisplatin resistance and radioresistance	103
SACC cell-derived EVs	/	Promote SACC cells migration and invasion	55

		proinflammatory cytokines, nerve growth factor	Educate human periodontal ligament fibroblast cells toward the protumorigenic phenotype	92
	CAF-derived EVs	MFAP5, ITGB1, BMI1, miR-34a-5p, miR-382-5p, miR-146b-5p, miR-3188, miR-196a, miR-876-3p	Promote the proliferation and migration of OSCC cells	93-101
**MSC-derived EVs**	BMSC-derived EVs	anti-inflammatory miRNAs, VEGF, miR-29a, miR-210-3p	Promote bone regeneration	120-121,123-124, 125-128
		miR-326	Promote cartilage regeneration	129-130,134
		miR-1246, lncRNA HCP5, miR-223-3p	Promote periodontal tissue regeneration	156-159,176-177
		miR-223, VEGF-A, FGF-2, HGF, PDGF-BB	Promote skin wound healing	183,185
		miR-101-3p	Inhibit OSCC progression	180
	UCMSC-derived EVs	VEGF-A, FGF-2, HGF, PDGF-BB, miR-21-5p, miR-125b-5p, Ang-2	Promote skin wound healing	186-189
	ADSC-derived EVs	VEGF-A, FGF-2, HGF, PDGF-BB	Promote skin wound healing	186

	miR-451a	Promote bone regeneration	122
DPSC-derived EVs	/	Promote bone regeneration	116-117
	miR-140-5p	Promote cartilage regeneration	131-132
	miR-150, miR-125a-3p, miR-146a-5p, miR-2110, miR-200b-3p, miR-223-3p, miR-1246, miR-494-3p, LOXL2	Promote pulp regeneration	17,140-141,143,146,148-150
	miR-378a	Promote periodontal tissue regeneration	170
	/	Promote skin wound healing	190
SHED-derived EVs	mitochondrial transcription factor A mRNA	Promote bone regeneration	118
	miR-100-5p	Prevent TMJOA	135
	miR-582-3p, miR-3622a-3p, miR-4657, miR-147b, miR-152-5p, miR-3152-3p, miR-7-1-3p, Wnt3a, BMP2	Promote periodontal tissue regeneration	166-169
	miR-100-5p, miR-1246	Inhibit OSCC progression	182

	PDLSC-derived EVs	miR-590-3p, miR-155-5p	Promote periodontal tissue regeneration	164-165,173-174
	SCAP-derived EVs	/	Promote pulp regeneration	142,147
		Cdc42	Promote tissue regeneration of palatal gingival complex critical-size defects	178
	GMSC-derived EVs	miR-1260b	Promote periodontal tissue regeneration	160-163
		IL-1RA	Promote gingival wound healing	179
		/	Promote taste bud regeneration	192
**Body fluid-derived EVs**	Salivary EVs	miR-24-3p, miR-512-3p, miR-412-3p, miR-302b-3p, miR-517b-3p, miR-140-5p, miR-143-5p, miR-145-5p, miR-10b-5p, miR-486-5p, miR-200a, Alix	Serve as biomarkers for the diagnosis of OSCC	195-198,202
		miR-125a-3p, miR-223-3p, miR-140-5p, miR-146a-5p, miR-628-5p, global 5mC hypermethylation, PD-L1 mRNA, CD9, CD81	Serve as biomarkers for the diagnosis of periodontitis	15,208-212

	miR-4484	Serve as biomarkers for the diagnosis of OLP	[14]
	miR-1307-5p	Serve as biomarkers for the prognosis of OSCC	[220]
Serum EVs	miR-130a, Alix, PD-L1 mRNA	Serve as biomarkers for the diagnosis of OSCC	[199-204]
	miR-let-7d, miR-126-3p, miR-199a-3p, miR-1304-3p, miR-200c-3p, SNORD57, SNODB1771	Serve as biomarkers for the diagnosis of periodontitis	[208,213]
	circ-IQGAP2, circ-ZC3H6, miR-127-3p, miR-541-3p, miR-409-3p, miR-410-3p, miR-329-5p	Serve as biomarkers for the diagnosis of SS	[218-219]
	miR-126, miR-130a	Serve as biomarkers for the prognosis of OSCC	[221,199]
Postoperative drainage fluid-derived EVs	EHD2, CAVIN1	Serve as biomarkers for the prognosis of OSCC	[206]
GCF-derived EVs	/	Serve as biomarkers for the diagnosis of periodontitis	[215]
PICF-derived EVs	miR-21-3p, miR-150-5p	Serve as biomarkers for the diagnosis of	[216]

			peri-implantitis	
	OKC fluid-derived EVs	/	Serve as biomarkers for the progression monitoring of OKC	207
**Tissue derived-EVs**	OLP/OLL-derived EVs	protein disulfide isomerase family A member 3	Promote the development of OLP and OLL	224
	Dental pulp tissue-derived EVs	/	Promote pulp regeneration	225

EVs extracellular vesicles, EBV Epstein-Barr virus, EV71 Enterovirus 71, SGEC salivary gland epithelial cell, SS Sjögren’s syndrome, OLP oral lichen planus, SASP senescence associated secretory phenotype, OSCC oral squamous cell carcinoma, CAF cancer associated-fibroblast, NPC nasopharyngeal cancer, SACC salivary adenoid cystic carcinoma, BMSC bone marrow mesenchymal stem cell, UCMSC umbilical cord mesenchymal stem cell, ADSC adipose-derived mesenchymal stem cell, DPSC dental pulp stem cell, SHED stem cell from exfoliated deciduous teeth, TMJOA Temporomandibular joint osteoarthritis, PDLSC periodontal ligament stem cell, SCAP stem cell from apical papilla, GMSC gingival mesenchymal stem cell, GCF gingival crevicular fluid, PICF peri-implant crevicular fluid, OKC odontogenic keratocyst, OLL oral lichenoid lesions

Supplementary Table 2. Three strategies for the preparation of artificial exosomes

Artificial exosomes	Cells or materials	Preparation strategy	Yield	Potential application	References
**Top-down strategies**	Monocytes or macrophage	Serial extrusion through filters with diminishing pore sizes (10, 5, and 1 μm)	100-fold	Deliver chemotherapeutic drug to treat malignant tumors	[227]
	Natural killer cell	Serial extrusion through filters with diminishing pore sizes (5, and 1 μm)	50-fold	As an immunotherapeutic agent for cancer treatment	[228]
	Murine embryonic stem cell	Slicing living cell membrane with microfabricated 500 nm-thick silicon nitride (SixNy) blades.	100-fold	Exogenous material delivery	[229]
	Human monocyte	Sonication	200-fold	Mitigate the symptoms of outer membrane vesicles-induced systemic	[230]

				inflammatory response syndrome	
	hUCMSC	Ultrasonication	20-fold	Skin regeneration and rejuvenation	[231]
**Bottom-up strategies**	/	Liposomes tailored with integrin α6β4	/	Targeted cancer drug delivery	[233]
	/	Liposomes bind with APO2L, TRAIL	/	Treat rheumatoid arthritis and haematological tumor	[234-236]
	/	Liposomes with membrane proteins chimeric modification	/	Targeted hepatocellular carcinoma therapy	[237]
	/	Conjugate a CD11c monoclonal antibody to the surface of immunoliposomes	/	Transdermal delivery of vaccines	[238]
**Biohybrid strategies**	Raw264.7 cell-derived exosomes and liposomes	Freeze-thaw technique	/	As nanocarriers for drug delivery	[239]

HEK293FT cell-derived exosomes and liposomes	Direct incubation	/	Deliver CRISPR/Cas9 system in MSCs	240
HUVEC-derived EVs and liposomes	PEG-mediated fusion	/	Efficient drug loading and delivery	241
Macrophage-derived sEVs and liposomes	Extrusion-based membrane fusion technique	/	Tumor-targeted drug delivery	242
CXCR4+ exosomes and liposomes	Extrusion	/	As an anabolic therapy for aged bone loss	243

EVs extracellular vesicles, MSC mesenchymal stem cell, UCMSC umbilical cord mesenchymal stem cell, HUVEC human umbilical vein endothelial cells

Supplementary Table 3. Strategies for the preparation of engineered exosomes by parental cell modification

Parent cell modification	Origin of EVs	Modification	Transfection vectors	Potential application	References
**Transfection**	MSC	yCD::UPRT	Retrovirus	Targeted cancer drug delivery	[244]
	OSCC cell	LncRNA ADAMTS9-AS2	Lentivirus	Suppress OSCC progression	[245]
	OSCC cell	LncRNA PART1	Lentivirus	Suppress OSCC malignant progression	[246]
	hADSC	miR-375	Lentivirus	Enhance bone regeneration	[247]
	BMSC	BMP2	Lentiviral particles	Enhance bone regeneration	[248]
	293T/17 cell	CXCR4 and miR-126	Lentivirus and RFect siRNA/miRNA Transfection Reagent	Alleviate periodontitis	[249]

	HEK293FT cell	NFIC	Lentivirus	Treat apical periodontitis and dentin regeneration	250
	HEK293 cell	miR-31	Lentivirus	Promote diabetic wounds healing	252
	MSC	HOTAIR	Plasmids	Enhance angiogenesis and wound healing	253
**Co-culture**	MSC	Paclitaxel	/	Package and deliver active drugs in cancer	254
**Preconditioning**	MSC	yCD∷UPRT and iron oxide (Venofer)	/	Facilitate targeted tumor cell ablation for tumor treatment	255
	BMSC	miR-21-5p	/	Enhance wound healing	256

EVs extracellular vesicles, MSC mesenchymal stem cell, BMSC bone marrow mesenchymal stem cell, ADSC adipose-derived mesenchymal stem cell, OSCC oral squamous cell carcinoma

Supplementary Table 4. Strategies for the preparation of engineered exosomes by direct exosome modification

EVs modification	Origin of EVs	Modification	Potential application	References
**Co-incubation**	HEK293T cell	Cholesterol-modified miR-34a	Inhibite OSCC cell proliferation, migration, and invasion	[263]
	HEK293 cell	exoSTING	Promote tumor immune surveillance	[264]
**Sonication**	Regulatory DC	TGFB1 and IL10	Treat degenerative alveolar bone disease	[265]
	Monocyte-derived DC	TGFB1 and IL10	Effective delivery system for immune modulation	[266]
	M2 macrophage	Melatonin	Inflammatory periodontal tissue regeneration	[267]
	M1 macrophage	Paclitaxel	Enhance the anti-tumor effects of chemotherapeutics	[268]

**Electroporation**	Normal fibroblast transfected with Epstein-Barr Virus Induced-3	siRNA of lymphocyte cytoplasmic protein 1	Attenuate oral cancer progression	269
	hADSC	miR-21-5p	Promote diabetic cutaneous wound healing	270

EVs extracellular vesicles, OSCC oral squamous cell carcinoma, DC dendritic cell, ADSC adipose-derived mesenchymal stem cell
